# “Like a Freshman Who Didn’t Get a Freshman Orientation”: How Transfer Student Capital, Social Support, and Self-Efficacy Intertwine in the Transfer Student Experience

**DOI:** 10.3389/fpsyg.2021.767395

**Published:** 2021-11-01

**Authors:** Rebecca Cepeda, Melissa T. Buelow, Shanna S. Jaggars, Marcos D. Rivera

**Affiliations:** ^1^Student Success Research Lab, The Ohio State University, Columbus, OH, United States; ^2^Department of Psychology, The Ohio State University, Newark, OH, United States

**Keywords:** transfer student capital, transfer, social support, self-efficacy, community college

## Abstract

Community colleges and other open-access two-year campuses provide an important pathway to higher education; however, a surprisingly small proportion of these students successfully transfer to and graduate from a bachelor’s degree-granting institution. The present study examined barriers and challenges students faced as they built their sense of self-efficacy as transfer students. We conducted interviews with 65 prospective or recent transfer students, including “internal” transfers (moving from an open-access predominantly two-year campus to their university’s flagship campus) and “external” transfers (moving from a community college to the university’s most selective campus). Our results indicate that both internal and external transfer students experienced challenges in terms of obtaining accurate information about transfer (transfer student capital, or “TSC”), but these challenges were easier to overcome for internal transfers, in part due to their social support networks. While both sets of transfer students utilized social support networks as an informal source of TSC, internal transfer students reported a more extensive social support network. Gaining accurate information about transfer and being supported by members of their social networks seemed to boost self-efficacy for transfer as well as adjustment during the post-transfer experience period. Recommendations for sending and receiving institutions are provided.

## Introduction

Each year, millions of students begin their pathway to a bachelor’s degree at a community college or other open-access predominantly two-year institution (Jenkins and Fink, 2020). Over half of college students attend a community college, and these students are disproportionately low-income, racially minoritized, or the first in their family to attend college (Rendón and Valadez, 1993; Ma and Baum, 2016; National Center for Educational Statistics [NCES], 2017). Although 50–80% of first-time community college students intend to transfer to a bachelor’s degree-granting institution, only about one-third ultimately do so; and among those who do transfer, only 42% complete a bachelor’s degree within six years of initial community college entry (Horn and Skomsvold, 2011; Jenkins and Fink, 2016; Wang, 2016).

For aspiring transfer students, the pathway from a two-year campus (the “sending institution”) through their destination four-year college or university (the “receiving institution”) may be influenced by multiple individual and institutional factors. In disparate strands of the literature, researchers have identified “transfer student capital” (TSC), self-efficacy, and social support as enablers of successful transfer (e.g., Laanan et al., 2011; Wang et al., 2017; Mobley and Brawner, 2019); however, few studies have examined the potential interplay between those factors during the transfer process.

In the present study, we explore the experiences of students from five open-access campuses (a large urban community college and four predominantly two-year university campuses) as they prepared to transfer to, or adjusted to a recent transfer to, a large public Midwest university’s flagship campus (MidU). We sought to examine the influence of TSC, self-efficacy, and social support on the transfer experience. In addition, due to the timing of data collection, we examined the extent to which these factors changed during the COVID-19 pandemic. The following sections outline the current research examining TSC, self-efficacy, and social support in the context of success in higher education more generally and for transfer students more specifically. We then briefly discuss the context of open-access predominantly two-year campuses such as those included in our study, and how that context may be similar or different from that of community colleges.

### Transfer Student Capital

Transfer Student Capital (TSC) refers to the knowledge accumulated at the community college or open-access institution that can aid in “vertical transfer,” or the transition to a more selective four-year institution (Laanan et al., 2011). TSC represents gained knowledge, including information about applications, financial aid, articulation policies, course curriculum alignment across campuses, and how to be a college student more generally (Townsend, 2008; Laanan et al., 2011; Moser, 2014; Lukszo and Hayes, 2020). The TSC framework builds upon the ideas of: (1) social capital, or the personal networks that provide individuals with both information and opportunities (Coleman, 1988; Moser, 2014; Mobley and Brawner, 2018), and (2) cultural capital, or one’s accumulated experiences within specific cultural contexts (Starobin et al., 2016). Although TSC is described in different ways in the literature to date, here we define TSC to include any information about transfer (i.e., when and how to leave a sending institution and enroll at a receiving institution) or post-transfer (i.e., how to acclimate to the receiving institution), whether that information was gathered through official institutional channels, other transfer students, or other sources.

TSC may be important to transfer student success due to the complicated and confusing nature of the transfer process. For example, some courses at the sending institution may not transfer to the receiving institution or may transfer as a general education or elective credit which does not count toward the student’s desired degree (Zeidenberg, 2015; Bahr et al., 2017; Belfield et al., 2017). Even when credits transfer seamlessly, course content may not align – that is, the sending institution course may not cover a topic that is critical to a sequenced course at the receiving institution – creating academic struggle in the second course (Packard et al., 2011). Even if a given pair of institutions has a high-volume transfer partnership, transfer students may follow more than one path to the receiving institution: some transfer after only one or two semesters of full-time enrollment, while others transfer after several years of intermittent or part-time enrollment, such that there is not an obvious, popular, “well-trodden” pathway to follow (Xu et al., 2018).

Students who need to develop TSC may leverage both formal and informal processes at both the sending and the receiving institutions. For example, TSC can be gained through interactions with peers, previous transfer students, friends, family, faculty members, high school staff, community college staff, and pre-transfer advisors; it can also be gained through coursework, pamphlets, websites, or other official sources (Townsend, 1995; Tovar, 2015; Laanan and Jain, 2016; Starobin et al., 2016; Jabbar et al., 2019; Mobley and Brawner, 2019; Rodriguez et al., 2019; Dang, 2020; Hayes et al., 2020; Jabbar and Edwards, 2020; Lukszo and Hayes, 2020; Schudde et al., 2020). Through these formal (institutional) and informal (social/cultural) sources, transfer students gain information necessary to navigate the transfer process, such as which courses to take at the community college in order to ensure seamless transfer of credits to their destination college, or when and how to complete an application to that college. In particular, peers (such as fellow community college and previous transfer students) can provide practical information which is unknown to or discounted by advisors or other official sources but is nonetheless vital to a successful transition and adjustment to the receiving institution (Flaga, 2006; Laanan et al., 2011; Moser, 2014). However, if the information provided by various sources is inaccurate or conflicting, students could become frustrated and confused (Ornelas and Solorzano, 2004; Allen et al., 2014; Walker and Okpala, 2017; Musoba et al., 2018; Schudde et al., 2021), or may lose time and money through excess credits or duplicated coursework (Laanan, 2003; Aragon and Perez, 2006; Owens, 2010; Bailey et al., 2013, 2015; Ellis, 2013; Jaggars and Stacey, 2014; Ngo and Kosiewicz, 2017). Collectively, these negative outcomes could lower students’ motivation and confidence in the likelihood of successfully persisting to a bachelor’s degree. Without the necessary support and TSC gained at the receiving institution that can affect academic self-efficacy, transfer students may be at higher risk of experiencing academic and social difficulties during their transition.

### Self-Efficacy and Transfer

Self-efficacy refers to an individual’s beliefs in their ability to succeed; and in the context of education, researchers often focus on academic self-efficacy, or beliefs that are specific to being academically successful (Bandura, 1977; Zimmerman et al., 1992; Bean and Eaton, 2002). High levels of self-efficacy, both academic and in general, are associated with greater academic achievement, retention, and overall GPA (e.g., Andrew, 1998; Bandura and Locke, 2003; Majer, 2009; Cardoso et al., 2013; Dutta et al., 2019; Travis and Bunde, 2020); engagement (Gutiérrez and Tomas, 2019); adjustment (Lee et al., 2018); and investment in goals and progress made toward them (Shulman et al., 2009). A recent meta-analysis indicated academic self-efficacy is one of the strongest predictors of academic achievement (Richardson et al., 2012). High levels of self-efficacy can also increase effort, persistence, and academically-oriented behaviors (Multon et al., 1991; Schunk, 1991, 2001; Bandura, 1997; Zepke et al., 2010), lower test anxiety (Kitsantas et al., 2008; Nie et al., 2011), and increase enrollment in challenging courses (Eccles et al., 1998).

Self-efficacy also seems to influence the transfer process. For example, Laanan et al. (2011) tested a model in which academic and social adjustment post-transfer were predicted by background factors, experiences at the sending institution, TSC, and experiences at the receiving institution. Negative experiences while trying to build TSC, such as inadequate advising support, lowered overall transfer experiences and post-transfer adjustment. Personal motivations for transfer, however, positively affected the experience and later adjustment. Although Laanan and colleagues did not explicitly measure self-efficacy, either in general or specific to transfer, self-efficacy can be altered based on positive and negative previous experiences. In a study of recent transfer students, Moser (2014) surveyed students about factors that predicted GPA post-transfer (i.e., at the receiving institution). Student motivation and self-efficacy were predictive, above and beyond background/demographic factors and experiences at the sending institution, and remained predictive even when factors associated with the receiving institution, such as transfer stigma and experiences with faculty, were factored in. In discussion of the results, Moser (2014) considered motivation and self-efficacy to be a critical component of TSC that can affect the transfer experience. In addition, higher levels of self-efficacy are also associated with a greater intent to transfer, improved transition experiences, greater academic achievement, and increased likelihood of degree attainment (Robbins et al., 2004; Dennis et al., 2008; Younger, 2009; Wang et al., 2017; Dang, 2020; Travis et al., 2020).

To more explicitly tie self-efficacy to transfer, Lukszo and Hayes (2020) described a type of self-efficacy specific to the transfer process which they termed “self-efficacy for transfer” (p. 43). Building from the work of Laanan et al. (2011) and Moser (2014); Lukszo and Hayes (2020) described how the process of building TSC through institutional and non-institutional sources can affect not just knowledge about transfer, but also the student’s self-efficacy to complete the transfer successfully. They pointed out that few studies to date specifically examine links between self-efficacy and transfer success, calling for future research to investigate not just what constitutes TSC, but where it is developed (e.g., institutional sources, peers, family) and how it affects the transfer process (e.g., self-efficacy for transfer).

Prior studies of self-efficacy and transfer have observed that higher levels of self-efficacy may be important to transfer success but have not examined how transfer self-efficacy is developed in the first place. Studies of general or academic self-efficacy in students suggest that these forms of self-efficacy can be built through previous successful experiences (Grether et al., 2018); relationships with parents, peers, and faculty members (Fan et al., 2009; Altermatt, 2019); and support from faculty and parents (Bandura et al., 2001; Ramirez et al., 2014; Alfaro et al., 2018). Thus, it seems likely that social support could also contribute to transfer-specific forms of self-efficacy.

### Social Support and Transfer

Social connections are a critical part of late adolescence and early adulthood (e.g., Erikson, 1959), with the most common sources of social support coming from family/parents and peers. In fact, support from family (Tao et al., 2000; Rayle et al., 2006-2007; Somers et al., 2006; Miller and Goldrick-Rab, 2015; Jabbar et al., 2019) and peers (Dennis et al., 2005; Rayle et al., 2006-2007; Somers et al., 2006; Whiteman et al., 2013; Miller and Goldrick-Rab, 2015; Altermatt, 2019) is associated with increased persistence, retention, and academic success for students. Support from college faculty members also improves academic outcomes (Tinto, 2012; Baker, 2013; Ayllon et al., 2019), and peer supports may have an even greater effect, particularly in terms of academic motivation (Dennis et al., 2005; Purswell et al., 2008; Baker, 2013).

Strong social support networks may also help students navigate the process of transfer. The experience of college transitions can be stressful, and there is some evidence that social support can buffer the negative effects of stress (Cohen and Wills, 1985; Gardner and Cutrona, 2004; Hefner and Eisenberg, 2009). In a study of first-generation transfer students, participants attributed their success in part to the support of their communities and felt the contributions of formal TSC were minimal in comparison (Mobley and Brawner, 2019). Dennis et al. (2008) found the greatest academic adjustment post-transfer among those with both high self-efficacy and high levels of peer support, with the lowest adjustment among students with low self-efficacy and low peer support.

Although social support may be important to the adjustment of transfer and non-transfer students alike, transfer students typically have a smaller social network at the new institution compared to their non-transfer peers (e.g., Harbin, 1997; Townsend and Wilson, 2006, 2008-2009), potentially increasing stress related to transfer and outcomes at the receiving institution. Transfer students also experience a higher level of stress and mental health concerns (e.g., depression, anxiety) more generally compared to their non-transfer peers (Beiter et al., 2015; Mehr and Daltry, 2016). In addition, “campus culture shock” (Archambault, 2015) can lead to perceptions that faculty and advisors are not supportive and difficulties forming new peer groups and accessing support services, all of which can negatively affect academic and social life (Davies and Casey, 1999; Flaga, 2006; Townsend and Wilson, 2006; Owens, 2010; Chrystal et al., 2013; Ellis, 2013; Jackson et al., 2013; Jorstad et al., 2017). Collectively, these prior studies suggest that transfer students benefit from social support, and experience difficulties with adjustment when it is absent.

### Integrating Transfer Student Capital, Self-Efficacy, and Social Support

In order to successfully navigate the transfer process and earn a bachelor’s degree, prior research suggests that students need to gain accurate knowledge about transfer, build networks of support, and be confident in their ability to succeed in this process. In the only prior study to examine all three components, Lukszo and Hayes (2020) suggest the three may also build upon and influence each other. In Lukszo and Hayes’ interviews of community college transfer students, interviewees reported developing TSC from peers, including previous transfer students, and community college advisors; these interactions not only provided TSC, but also provided boosts to students’ confidence and self-efficacy for transfer. Gaining knowledge from supportive individuals boosted the students’ self-efficacy to succeed, in part by helping them prepare not just for the transfer itself, but for academic life at the receiving institution. However, Lukszo and Hayes’ study represents one of the first investigations of self-efficacy for transfer, and further investigations of the interplay between these factors is warranted.

TSC, social support, and self-efficacy likely interact with one another to influence the transfer process and adjustment to the receiving institution post-transfer; however, little research to date has examined this interplay. Transfer is stressful and experiencing a significant stressor can decrease academic adjustment (Ruberman, 2014). Theoretically, embarking on a stressful event with significant gained knowledge (TSC) and the support of others could boost self-efficacy, which in turn could increase task persistence and motivation (Wang et al., 2017). In this paper, we will examine how TSC, social support, and self-efficacy influence one another during the transfer process, and the extent to which gaps in an efficient and supportive transfer process may negatively influence TSC, support, and self-efficacy.

### Open-Access Predominantly Two-Year Campuses

The literature on community college student success is broad and deep, including the back catalogs of entire journals such as *Community College Review*; the topic of transfer from community colleges to four-year colleges is also well-explored (for a broad overview of key research findings, see Jenkins and Fink, 2015). In comparison, researchers have largely ignored a similar sector of colleges: open-access campuses of larger university systems, which accept most or all applicants who wish to enroll at the campus. While some open-access university campuses offer a full array of bachelor’s degrees, other “predominantly two-year” campuses require students to transfer to a larger campus to complete some or most of the university’s majors. These campuses are rarely-studied; indeed, we are aware of *no* studies which specifically focus on this sector, although these institutions are sometimes included in larger studies of open-access campuses (e.g., Rutschow et al., 2019). The lack of attention to these campuses may be due to their lack of clear definition: the federal Integrated Postsecondary Education Data System (IPEDS), which is the nation’s authority on the number, type, enrollment sizes, and outcomes of United States colleges, makes no clear delineation between community colleges which offer a small selection of bachelor’s degrees, predominantly-two year campuses of larger four-year systems, and other four-year campuses which award a mix of associate and bachelor’s degrees (see Fink and Jenkins, 2020). As a result, we have no national estimate of the prevalence, enrollment sizes, or student success outcomes of predominantly two-year regional campuses such as those included in this study. Yet at least in the university under study, predominantly two-year regional campuses rival community colleges in terms of the volume of transfer students (including first-generation, low-income, and underrepresented minority students) they send to the most selective campus each year.

### The Present Study

The present study sought to examine experiences of students transferring from community colleges and open-access institutions to a selective four-year campus, focusing our investigation on the influences of TSC, social support, and self-efficacy. Although previous research indicates the importance of TSC, social support, and self-efficacy as separate factors in the transfer process, few studies outside Lukszo and Hayes (2020) specifically examine factors that affect self-efficacy for transfer and how this can affect the transition. In addition, most studies to date focus on community college transfer, with little known about the transfer process from open-access predominantly two-year university campuses to a more selective campus. In the present interview-based study, we examine how TSC, self-efficacy, and social support affect the transfer experience, both pre- and during the COVID-19 pandemic. We employ a narrative inquiry approach to understand individual experiences and perceptions of the transfer process, as well as the broader social, cultural, and institutional narrative in which their experiences are situated (Clandinin and Connelly, 2000; Chase, 2011). We assessed the following study aims:

(1) Examine how TSC, self-efficacy, and social support each affect the transfer process, assessing how they change as a function of barriers or challenges for students interested in transfer;
(2) Examine how TSC, self-efficacy, and social support influence each other during the transfer process, as well as how challenges in one area affect development in other areas;
(3) Examine the extent to which transfer experiences and these influencing factors vary across community college and open access sending institutions; and
(4) Assess how the COVID-19 pandemic affected transfer students’ experiences.

## Materials and Methods

### Study Setting

This study focuses on a large selective flagship campus in the Midwest (MidU) which welcomes over 3,500 new internal and external transfer students each year. Internal transfers arrive from one of the university’s four open-access regional campuses (hereafter termed simply as “regional campuses”), which are located in rural or small-town areas of the state. While these campuses offer a small selection of bachelor’s degrees, the vast majority of students must transition to the flagship campus to complete their intended degree. To apply for internal transfer, regional students submit a “campus change” request, which is typically approved once the student has earned at least 30 college credits with a minimum GPA of 2.0. Because the university’s campuses use a common course numbering, degree audit, and advising system, regional campus credits are automatically and seamlessly applied when students transition to the flagship.

External transfers typically arrive from a nearby large urban community college (Nearby LUCC). Students from Nearby LUCC and all other in-state community colleges are guaranteed admission to the flagship campus if they have earned at least 60 credits with a minimum GPA of 2.0, although they are not guaranteed admission into specific majors. To ease the transfer process, Nearby LUCC uses the same course numbering system as MidU. Courses offered by both institutions are transferred seamlessly; however, Nearby LUCC offers some courses not offered by MidU, and these courses may receive greater scrutiny before determining whether or how they will be applied to the transfer student’s degree program.

The three types of institutions differ sharply in the list price for in-state students, with Nearby LUCC charging the least (around $4,500 per year in 2019–2020), the regionals charging more (around $8,000), and the flagship charging the most (around $11,000). However, Pell-eligible students’ tuition and fees are waived entirely at the flagship and are waived starting the second semester of enrollment for regional campus Pell-eligible students.

### Recruitment and Procedure

Analyses focus on *N* = 65 interviews with prospective or recent transfer students from sending institutions. Two subsets of students are included in the sample. First, we partnered with administrators from each institution to disseminate interview recruitment e-mails to students who were interested in transferring to MidU, which yielded *N* = 18 participants from local community colleges. Thirteen of those participants were enrolled at Nearby LUCC and five were enrolled at other community colleges with a similar profile as Nearby LUCC. For the purpose of this paper, we collectively refer to these community college institutions as Nearby CC. Additionally, we interviewed *N* = 17 students enrolled at regional campuses who intended to transition to MidU. Second, students who already transferred to MidU from one of these institutions were identified through institutionally held student record data. From this list, we generated a stratified purposeful sample of students who transferred to MidU within the last year to include a relatively even mix of students from different academic programs, campuses, demographic profiles, and transfer statuses. We emailed students with an invitation to participate in the study and interviewed *N* = 21 participants who had transferred from Nearby CC and *N* = 9 who had transitioned from a regional campus. MidU is a Predominantly White Institution, so a majority of the students interviewed were White; approximately a quarter were Students of Color. Students were enrolled in a wide variety of majors, including engineering, communication, linguistics, and animal science.

Students were offered a small monetary incentive to participate in an interview, which took place between Fall 2019 and Summer 2020. One-on-one interviews of up to one hour were conducted virtually or in-person using a semi-structured protocol. The protocol was built upon the methods and findings of previous qualitative research on community college students’ experiences with transfer (particularly Wyner and Jenkins, 2017; Jaggars et al., 2019). The interview protocol explored why a student decided to attend an open-access institution, their rationale for transfer, and their experiences throughout the transfer preparation and transition processes. Interviews were audio-recorded and transcribed by a professional transcription service. A total of *N* = 65 interviews were conducted, including *N* = 51 interviews prior to COVID-19 campus closures and *N* = 14 interviews during COVID-19.

### Analysis

**FIGURE 1:**
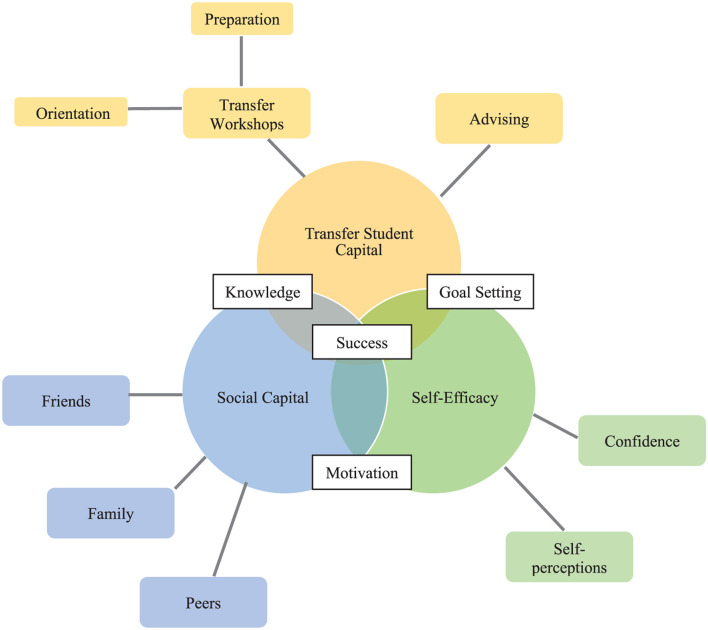
Thematic map, showing the interconnections between Transfer Student Capital (TSC), social capital, and self-efficacy and its relation to transfer student success.

We employed a theoretical thematic analytic approach, in which our analysis was driven by our theoretical interest (Braun and Clarke, 2006) to understand the TSC transfer students receive at both their sending and receiving institutions. This included a two-stage process. First, transcripts were analyzed by assessing themes and developing a preliminary set of codes. Codes were based on the transfer process and student perceptions of the process. We utilized a semantic focus on coding, or “coding and reporting on explicitly-stated ideas, concepts, meanings, experiences…” rather than latent themes that entail analysis to be developed through implicit ideas (Braun et al., 2016, p. 193). We used the preliminary codes to analyze an initial subset of data and then refined codes based on their usefulness in capturing key themes we identified from the data, using TSC as a lens to focus our understanding of the complex set of transfer process and experiences that students articulated (Laanan and Jain, 2016). We defined self-efficacy as statements that reflect questions about the sense of self or self-confidence. We defined social support as support received outside of formal institutional services, from students’ social network such as family, peers, and friends. Based on prior studies of TSC, we defined it as statements dealing with logistic issues around transfer, such as when or how to perform specific transfer-related tasks. However, for each of the *N* = 30 post-transfer interviewees, the three constructs carried slightly different meanings during the pre- versus post-transfer period. After transfer, TSC morphed from knowledge about the transfer process itself, to knowledge about how to navigate the complexities of MidU as a new transfer student. Similarly, transfer self-efficacy shifted toward the student’s sense of being a successful MidU transfer student. The research team reviewed and refined the codes to capture shared themes. See Figure 1 for an example of how our thematic map demonstrated the interconnections between TSC, social support, and self-efficacy. Once an agreed-upon coding scheme was finalized, coded transcripts were further analyzed to identify patterns in the data (Creswell and Poth, 2018).

## Results

Below, we first provide a general overview of why interviewees chose to enroll in either a regional campus or community college, and the types of self-efficacy and social support they initially brought into college. Next, we discuss the pre-transfer experience, including how students built TSC in conjunction with social support and transfer self-efficacy. We then consider the post-transfer experience, including the ways students continued to acquire TSC, social support, and transfer self-efficacy. Finally, we discuss how COVID-19 and the related campus closures influenced students’ social support and transfer self-efficacy.

### Initial Entry Into College

Prior to enrolling in any college, internal and external transfer students had relatively similar academic experiences. Typically, both groups had long aspired to attend MidU’s flagship campus, but despite performing well in high school, their academic record was not sufficiently competitive to gain direct admission into the MidU flagship. Most interviewees who chose to enroll at a regional campus saw it as an efficient and straightforward path into the flagship, and typically planned to transition immediately upon earning 30 credits; however, a few regional campus interviewees felt a smaller and more close-knit campus would provide a gentler introduction to college life and planned to stay at their campus for longer before transitioning to the urban flagship. For example, one internal transfer student expressed:

I felt that it was a really good way to kind of ease into college and ease into that transition. Starting at a bit of a smaller campus just because main campus is so large and it’s kind of a shock to go from high school to that.

While some regional campus students already lived in the local area, others moved to live on or near a regional campus or commuted long daily distances from the city.

Interviewees viewed the regional campus experience as an opportunity to “prove” themselves, using language that resonates with the self-efficacy framework. For instance, internal transfer students who were not initially accepted to the flagship institution described their motivation to pursue the transfer process. One student expressed:

My ACT scores were a little low so I had to attend the regional campus…I had to go into the regional campus with a mindset of leaving…a goal of being there for a year to prove myself that I can actually compete in the main campus.

Upon their arrival at the regional campus, interviewees typically had few concerns about their ability to eventually succeed and transition to the flagship; their primary concern was focused on the increased tuition or living costs that would be required after the transition, and this worry did seem to undermine some students’ self-efficacy. For example, one transfer student from a regional campus expressed:

I was very nervous as to how that would translate to [MidU] since it is a big change in cost. Just making sure that I was ready to be able to not only afford to go to school, but afford to pay for my own living, since the cost of living is much higher here. Another was whether or not I was ready. I love the environment [at MidU], but it is still a lot for me.

Concerns about the increased costs associated with transitioning to the flagship, both in terms of educational costs and living costs, seemed to temper regional campus students’ self-efficacy for transfer, raising self-doubts about the decision to transition and success afterward.

On the other hand, interviewees who chose Nearby CC explained that it was nearby and low-cost, while still offering a fairly straightforward pathway to the MidU flagship; most planned to transfer soon after earning 60 credits. As one Nearby CC student explained:

I came to [Nearby CC] because I am providing for myself for education… so I thought it was easier for me to just come to [Nearby CC] because it’s a lot cheaper than [MidU]. At least [Nearby CC] do offer transfer programs, where I can just go to [MidU] after and they’ll help me out with the steps and stuff…yeah, that was my number one goal, was to transfer to [MidU].

In comparison to regional campus students, Nearby CC students were less likely to discuss a desire to prove themselves and more likely to discuss logistical considerations, such as the need to save money while they worked on boosting their GPA or deciding on a major. One external transfer student stated:

I couldn’t afford to go to the other colleges I applied to, and because I wasn’t sure about what I wanted to study quite yet, I decided to just take some classes and just kind of explore it over there a bit more before like putting full…focus into like a four-year university or a bigger university. So that was like the main thing is I wasn’t sure exactly what I wanted to do, so going to a community college was a better way to try and figure that out.

The student endorsed both financial reasons for starting at a community college, as well as reasons related to boosting self-efficacy for major choice, rather than transfer-specific self-efficacy.

At initial entry into the regional or community college campus, students already differed in the extent to which they had social ties with MidU. Regional campus students were more likely to report knowing family or friends who were currently enrolled at MidU, previously attended MidU, or worked at MidU. Although some Nearby CC students indicated knowing friends and roommates recently or currently attending MidU, they placed less emphasis on these ties in comparison to regional students.

In general, regional campus students seemed more eager to switch to the flagship campus as quickly as possible, while community college students seemed more willing to delay their entry into the flagship campus in order to gain a few more semesters of affordability or flexibility.

### Pre-transfer Experiences

Prior to transfer, all five sending institutions explicitly worked to help students develop TSC through formal approaches, a process which was reinforced for students who had higher levels of social support. In turn, students with strong TSC and social support seemed to accumulate higher levels of self-efficacy as they moved toward transfer.

*Formal provision of TSC.* Generally, both Nearby CC and the regional campuses offered formal sources of TSC such as transfer workshops or one-on-one transfer advising. For instance, one internal transfer student reported that institutional agents at their regional campus highly promoted the internal transfer process:

The advisors [at the regional campus] were awesome…they left flyers around campus to make sure that we knew when the [transfer] meetings were. Having [my advisor] as a middle person…she always told me if…I had any questions, to e-mail her. She had given me her phone number for her office…that was super helpful because any questions I had, I sent them to her. Then there was a meeting where [she] gave me an information packet telling me how [the transfer process] was like and what I should expect [after I transferred].

This advising meeting helped the student understand what classes they needed for transfer to MidU. The student was now able to envision their future at MidU and understood the next steps needed to achieve their transfer goal.

Although institutional support for transfer was available at all five sending institutions, Nearby CC students were less likely than regional campus students to engage with such supports. Community college interviewees typically had time-consuming external obligations, such as working off-campus or supporting their families, and thus were less likely to become involved in campus supports and other activities. Some transfer students were unaware of institutional transfer supports or unsure of how to access them, and instead worked to “figure it out” on their own. One student shared:

When I went into [Nearby CC], they had a couple [pathway programs] but my major didn’t have any pathway programs, so I had to figure out on my own what classes to take… I didn’t really know whether or not [classes] would transfer over correctly or what classes for sure I needed. I managed to find the classes I needed on [MidU’s] website…it was like buried somewhere. I don’t even remember where I got it from, but I had it and I went off of that, mostly.

Although this transfer student utilized passive institutional support, like the university website, to navigate the courses needed for transfer, they were still uncertain whether their courses would successfully articulate to MidU; without more personalized forms of support, they were lost and unsure how to proceed.

Among Nearby CC interviewees who did seek support from institutional agents regarding transfer, most felt the support they received was inadequate. One student reported that Nearby CC’s transfer center was under-resourced, and primarily employed part-time staff as transfer advisors or offered information sessions at times which conflicted with the student’s class or work schedules. They expressed their unsatisfactory experience with the transfer support staff at their sending institution:

The administrators are very difficult to talk to [at the transfer center], and so I’ve had a few difficulties trying to have them help me with my classes—what classes to pick—and they’ve had a lot of difficulties…my [transfer support] administrator is part time, so I get bounced to everyone instead.

While this student attempted to obtain TSC from institutional agents, they never received a clear understanding of requirements for transfer. Ultimately, they still persevered, applied, and transferred to MidU; however, they felt the process was more stressful than it should have been.

*Social support: an informal source of TSC.* In addition to formal sources, Nearby CC and regional campus students also acquired TSC through their social networks; however, the relevant social contacts were broader and deeper for regional students. In comparison to Nearby CC students, it seemed much easier for regional campus students to build social support networks within the relatively close-knit small-town campuses. Regional students indicated that small class sizes supported their academic growth as students and helped them become comfortable reaching out for support. As a result, they were more inclined to seek an on-campus job, make connections with faculty members, and cultivate friendships with their peers. In turn, a wide and deep social network provided a rich informal source of TSC. In contrast, at the large urban Nearby CC, students’ off-campus work and family responsibilities constrained them from becoming involved with student organizations and cultivating on-campus relationships to the extent they would have liked.

Social support from family and friends who had already graduated from college, transferred to MidU, or were currently enrolled at MidU, were particularly helpful for both regional and community college students as they tried to understand the process of transfer. One Nearby CC student described how impactful it was to learn about the process from a peer who already transferred:

She basically was like, “this is how you [transfer to MidU] …this is what you’ll need…these are the ABCD steps,” which made it kind of easier for me because I already knew what to expect as far as applications and the fees and this and that. So, I kind of already knew like “this is what’s going to happen, this is going to happen next.”

Guidance from friends and family helped students create a clear transfer roadmap that clarified due dates, fees, and applications, and strengthened students’ TSC.

*Reinforcing transfer self-efficacy.* In addition to purely informational value, social networks and TSC helped students build transfer self-efficacy. Seeing people that they personally knew successfully transfer was a critical component to students’ own sense of motivation and self-efficacy. As a regional campus student was planning to transfer to MidU, they shared their sentiments on how it felt seeing friends who attended the same regional campus complete the process:

Honestly, just thinking “…I’m changing campuses next fall.” That can be so overwhelming…and just seeing how [my friends] went through it…they didn’t have any issues; they’re doing just fine. Look at them, they’re out there, they’re having a great deal of fun, they’re involved, they’re doing good in the classroom. That makes us feel so much more confident when we can look at them and say “Okay, I can do this too.” I hear all good things, and it makes me feel confident that I know I’ll have the same experience transitioning.

In general, transfer narratives from students’ social networks both strengthened TSC and served as a catalyst for students to persist with their transfer process and transition.

*Summary of the pre-transfer experience.* When students obtained TSC from formal institutional sources or informal social sources, they leveraged the knowledge they acquired, gained a stronger sense of self-efficacy, and felt more confident in asking clarifying questions and preparing for the logistical process of transfer. Informal social networks were also particularly useful for increasing transfer students’ self-efficacy: seeing a friend succeed provided a sense of confidence for the students’ own transfer process as well. However, regional students seemed to have greater access to both formal and informal sources of TSC than did community college students, which seemed to be due to the regional campuses’ smaller sizes, their students’ relatively strong engagement with on-campus life, and the sheer volume of fellow students following the same pathway to MidU.

### Post-transfer Experiences

After matriculating at MidU, both internal and external transfer students shared similar themes in how they continued to accumulate TSC from both formal and informal sources within their new campus environment.

*Formal provision of TSC.* In terms of formal sources, MidU advisors were commonly cited. For example, one Nearby CC transfer student expressed how helpful their MidU advisor was in terms of supporting their transition:

I had my first meeting with my advisor, and she’s just amazing. Like she laid everything out. She selected my first courses…I haven’t had a bad recommendation from her yet, and she really helped walk me through the whole process while being here. As soon as I got connected to her, everything went smoothly. I wanted to do an undergraduate thesis. She helped me set all that up. She took care of my graduation and stuff because I had a bit of a weird path that I took…as soon as I got in contact with [my advisor], nothing was difficult.

Advisors not only provided TSC in terms of how to manage the immediate transition process, but also acted as a form of social support that reassured students that they could achieve long-term success at MidU.

However, even after transfer, students faced challenges with adjusting to a new campus. Both internal and external transfer students expressed feeling like a “glorified freshman” and experiencing challenges with primarily non-academic logistics. For example, students often voiced that they felt lost while navigating a larger campus and were unsure of where or who to ask for support—as if they were a first-year student. They felt people assumed they should already know how to be successful at MidU because they had “already been to college.” One internal transfer student recounted:

I felt like when I got [to MidU’s campus], it was really confusing. There’s nobody showing me around campus. Granted, I’m an adult. I could have done it myself, and I did. But it seems like out here at main campus…everybody’s like, “Well, here you go.” And I didn’t really know where anything was at or anything like that. Like a freshman who didn’t get a freshman orientation.

In general, both internal and external transfer students felt MidU did not provide enough formal resources to help them develop the TSC that would have facilitated success once on campus.

*Social support: an informal source of TSC.* After transferring to MidU, both internal and external transfer students reported developing TSC from social support networks, such as friends who previously transferred, those who started at MidU as freshmen, and cultural and identity-based student organizations. For example, an internal transfer student shared that they depended on their friend to learn how to navigate the large campus:

My best friend…we went to high school together…we played soccer together. [After high school] I went to [a regional campus] and she went to [MidU’s] main campus…I knew if I was lost, I could call her and say, “Oh my gosh, where am I?” She helped me a lot…figuring out the little things…like if I’m going to be late to office hours, I [should] e-mail…stuff like that.

Supports from friends and peers already at MidU were helpful for both internal and external students’ social transitions such as navigating their way around campus, finding a place to live, or learning about campus organizations. Tapping into an active support network also helped students clarify and ease potential challenges, most notably when TSC was not gained in that area.

For internal and external students who reported not having a social support on campus, creating academic networks on a new campus was challenging. An external transfer student shared their experience trying to find a community of support once they arrived to MidU:

It was really hard for me to find study friends or go out of my way and talk to people that I didn’t have my freshman and sophomore year within the dorms. I didn’t create those kinds of connections. I’m just coming in as someone who’s like “some random [person],” and it was really hard to talk to people, to get to know people, to develop those study buddies, and like people to work with together. So, that was the most difficult thing for me…that was the hardest part of the transition, just finding people to study with, because people don’t want to talk to people here.

In contrast to the robust “first-year experience” programming integrated into the everyday experiences of incoming freshmen, MidU lacked processes and infrastructures for facilitating social connections among transfer students. For example, interviewees identified a lack of a physical space or campus organizations specifically designated to help cultivate a transfer student community:

It would be probably extremely helpful if there was a [transfer] club…or organization that would take you aside before orientation or…before school started…to give [transfer students] an opportunity with doing different things…going to different events, making friends if you don’t have friends when you come here…

Despite being disappointed and frustrated by a lack of institutional support throughout the transfer and transition process, transfer students pulled on their own tenacity and self-motivation to continue to push forward. One student even shared, “overall, [my transfer experience] was fine, but again, I think it was mainly just because I’ve had to do everything on my own since I was younger.”

As noted in an earlier section, during interviews with pre-transfer students, regional students were more likely than Nearby CC students to mention social contacts who had already transferred to MidU. Yet in interviews with post-transfer students, both internal and external transfers were equally likely to mention social contacts at MidU and emphasize their importance. Regardless of whether they were internal or external transfers, social supports helped students build their TSC and sense of self-efficacy as a successful MidU student. However, some transfer students who did not have an existing social support at MidU found it challenging to connect with peers to form study groups or create academic relationships, which may have negatively impacted their self-efficacy. For instance, a Nearby CC student went into detail about how social support would have been helpful to their success at MidU:

The only initiative [MidU] took…helping students transition…wasn’t even really a social [initiative]. That was more or less just like, “Let’s make sure you know what you’re doing academically for your time here.” But I think it’s hard not to think about [social support] because it’s such an important part of [school]…like feeling connected to the school. I think it’s a really important part of success because you can’t really separate it…From my experience, it was this disconnectedness that has been pervasive throughout my time here. I don’t feel connected to [MidU] at all.

Although MidU made efforts to support transfer students with academic issues, it did not create space for facilitating their social connections, which is a part of their academic success. Overall, these examples demonstrate that the acquisition of TSC, social support, and self-efficacy is fundamental for transfer students across both pre- and post- transfer environments.

### Impacts of COVID-19

During the Spring 2020 semester, MidU, the regional campuses, and Nearby CC were forced to switch to remote learning and student services due to the COVID-19 pandemic. In general, the challenges transfer students faced were amplified due to the transition to virtual instruction. Students who had already transferred to MidU experienced yet another academic transition, in turn lowering their self-efficacy and motivation toward their academic progress and potentially impacting their future academic goals. One student described their experience with transitioning to remote learning and the loss of motivation with their coursework:

I feel like I could’ve done better in certain classes, had I been on campus…the final grades I ended up with in a few classes may influence my likelihood of getting into a [graduate school] program.

The transition to virtual instruction dampened the student’s motivation to sustain their academic progress, which may have lasting impacts in terms of their aspirations to attend graduate school. Additionally, the uncertainty of academic progress toward a career path even compelled some students to think about taking time off from school. Another student suggested they had concerns with taking their major classes remotely:

One thing that I think has made me step back and wonder like, “Should I just maybe wait a year to take these [art] classes?” Because I want to make sure I’m getting everything out of the class so I can be the best art teacher…

In general, being away from a physical educational environment and social support seemed to decrease students’ self-efficacy and add challenges to their academic and career goals.

Moreover, virtual educational experiences exacerbated the challenge of cultivating genuine relationships and friendships with peers. As one external transfer student explained:

It’s a lot harder [to attend class and make friends online]…you can’t get out of your bedroom…and have family or roommate distractions…so you can never go back…and ask your classmates, “What did the professor even say?”

Due to this challenge with forming relationships with peers, both internal and external transfer students expressed that MidU should create a virtual setting for peers to interact with one another. Overall, the challenges transfer students experienced from the switch to virtual instruction due to COVID-19 were similar to those pre-COVID-19, but were even more heightened, as transfer students continued to find ways to acclimate into the new virtual campus environment at MidU.

## Discussion

In the present study, we examined barriers and challenges to the internal and external transfer process in the context of three key factors: transfer student capital (TSC), social support, and self-efficacy for transfer. To date, literature on the transfer student experience has primarily focused on transfers from community colleges to four-year bachelor’s degree-granting institutions. We expanded this perspective by examining two types of transfer students: external transfer students from nearby community colleges, and internal transfer students from open-access predominantly two-year regional campuses.

Across the two types of transfer students, our results suggest that while internal and external transfer students share some of the same challenges and difficulties during transfer, internal transfer students have some logistical benefits that external transfer students do not. Internal transfer students expressed fewer concerns about academic logistics, such as course articulation policies and credit transfers, than external transfer students, likely due to common course offerings and advising systems across campuses. Although Urban LUCC utilizes the same course numbering system as MidU, not all its courses are offered at MidU, which can negatively impact those courses’ transferability. External transfer students expressed greater concerns than internal transfer students about credit transfer and access to formal institutional supports, such as academic advisors, to whom they could ask these questions.

A second factor that differed across internal and external transfer students, was the extent of informal sources of TSC prior to transfer. At initial college entry, internal transfer students were more likely than external transfer students to know someone currently or previously at MidU. Although both utilized their social support networks as informal sources of TSC pre-transfer, external transfer students were less likely to report being engaged with the sending institution community or with previous transfer students; instead, they reported figuring out the transfer process on their own. This difference in the extent of informal TSC networks is concerning, as both internal and external transfer students spoke to the importance of seeing someone they know successfully transfer, and how that can positively affect transfer self-efficacy. We saw fewer differences in social supports among our post-transfer interviewees; however, it is possible we observed this pattern because community college students who lack these informal TSC networks (in particular, connections with successful transfer students at MidU) fail to successfully transfer to MidU.

Despite differences in the pre-transfer experience, internal and external transfer students expressed similar positives and negatives at MidU. Both types of students reported relying on formal sources (advisors) for academic logistical concerns, and both expressed concerns about the lack of opportunity to develop informal support networks for both academic and non-academic concerns. The COVID-19 pandemic shed even more light on the importance of informal social support networks to boost transfer self-efficacy and motivation. After classes and transfer-related programming (including orientation) transitioned to a virtual format, students lamented losing the informal support and knowledge gained from conversations with other students. It became even harder to build a sense of community at the receiving institution, lowering student motivation and self-efficacy not just for academic success at MidU but for their overall career goals as well.

Collectively, our findings suggest several implications and considerations for sending and receiving institutions to improve the transfer student experience. Although much of the transfer literature to date centers on sending institutions, receiving institutions also have a responsibility to support successful transfer (Bryant, 2001; Jain et al., 2011; Gandara et al., 2012; Bahr et al., 2013; Handel, 2013). Working in tandem, sending and receiving institutions can work to create a “best case scenario” for transfer success, in which the student gains accurate knowledge about transfer, has a network of peers who are on a similar path, and feels confident in the transfer process.

First, both sending and receiving institutions can simplify the level and amount of information that students must access, process, and execute in terms of transfer, by aligning pre-requisite courses across campuses, solidifying articulation agreements, and developing academic plans to set students up for success; they can also ensure students are given accurate information early in the transfer process, and train both sets of advisors on transfer-related policies and procedures (Wright and Middleberg, 1998; Berger and Malaney, 2003; Handel, 2007; Jain et al., 2011; Packard et al., 2011; Lopez and Jones, 2017). The value of simplifying the process is clear when we juxtapose internal and external transfers in this study: Internal transfer students, for whom the credit transfer process was seamless, expressed less concerns about credit transfer to MidU than external transfer students, which also served to boost their self-efficacy for transfer.

Second, institutions can increase opportunities for aspiring or recent transfer students to meet successful post-transfer students. Fostering these informal support networks pre-transfer can boost self-efficacy for transfer, as students will have additional sources of knowledge that they can tap into during transfer. Post-transfer, institutions can provide a transfer-specific orientation which intentionally includes opportunities for peer networking, can create a transfer student organization or dedicated space on campus, and can facilitate connections between current transfer students, previous transfer students, and students who started at the receiving institution, perhaps through a peer mentoring program (e.g., see Flaga, 2006; Owens, 2010). Transfer students who have high levels of self-efficacy may be more likely to share knowledge with other transfer students (e.g., Ergun and Avci, 2018); thus improving one student’s transfer process can pay dividends for the future students who benefit from this new source of social support.

Creating additional opportunities for students to gain TSC, develop social supports, and boost self-efficacy for transfer and academic success can improve the transfer experience for both internal and external transfer students. Offering both in-person and virtual orientation and transfer-related events may provide additional opportunities to succeed for those students with significant external family and work obligations that limited attendance at solely in-person events pre-COVID.

### Limitations and Future Directions

This study has several limitations. First, in terms of race and ethnicity, the key findings of this study seemed fairly consistent across participants of different races. However, our study protocol did not include specific questions about how race, ethnicity, or other important identities may have influenced students’ experiences as college students or transfer students. If a similar study was conducted in the future, we would suggest asking more explicitly how particular identities may have influenced the transfer process, as this is an important area for future study given the changing societal lens regarding identities. Second, MidU has strong articulation agreements, common course numbering, and high rates of transfer with the sending institutions under study. This infrastructure created clearer and smoother pathways than is typical of most sending-receiving pairs. Thus, our findings might understate the challenges inherent in transfer for many other students across the country. Lastly, not unique to this study but worth mentioning, the current landscape of higher education and transfer have been complicated by the COVID-19 crisis. Findings and research conducted before the coronavirus is still useful, but consideration of context is particularly warranted as the lasting implications of the pandemic are still unknown.

### Conclusion

Our findings indicate that students with active sources of TSC experienced an easier transfer process, whereas those without social and academic networks at both the sending and receiving institution felt less confident in their choices, unsupported, and in some cases, discouraged from belonging to the new institution. However, these students still showed determination and were motivated to complete the transition and persist to graduation as best as they could, highlighting the importance of considering intrapersonal resources such as self-efficacy in addition to social and cultural capital (Jabbar et al., 2019).

This study is significant because it suggests that internal transfer students who transition to a more selective campus struggle with many of the same challenges that have been previously documented among community college transfer students; however, institutional design choices may make it easier for these students to navigate those challenges. By examining students’ experiences both prior to and after transfer, the study also provides a more thorough perspective of the entire process, not only pre-transfer challenges (Handel and Williams, 2012). Additionally, we obtained knowledge about the experiences of transfer students amidst the COVID-19 pandemic and the transition to remote learning and student services. Student stories can inform administrators and other institutional agents at open-access institutions and four-year institutions of the success and gaps identified by these narratives. Findings can encourage the formation of partnerships and collaboration across institutions to develop policies and practices to better support transfer student success.

## Data Availability Statement

The datasets presented in this article are not readily available because this study was primarily qualitative in which the investigation relied heavily on in-depth one-to-one interviews with participants. In order to maintain confidentiality and not share participants’ identifiable data, we cannot share interview transcripts. Requests to access the datasets should be directed to SJ, jaggars.2@osu.edu.

## Ethics Statement

Ethical review and approval was not required for the study on human participants in accordance with the local legislation and institutional requirements. The patients/participants provided their written informed consent to participate in this study.

## Author Contributions

SJ conceived of the study and its design, obtained funding, supervised recruitment, coded and interpreted data, and revised the manuscript. MR recruited and interviewed participants, coded and interpreted data, and revised the manuscript. MB and RC recruited and interviewed participants, coded and interpreted data, and wrote and revised the manuscript. All authors contributed to the article and approved the submitted version.

## Conflict of Interest

The authors declare that the research was conducted in the absence of any commercial or financial relationships that could be construed as a potential conflict of interest.

## Publisher’s Note

All claims expressed in this article are solely those of the authors and do not necessarily represent those of their affiliated organizations, or those of the publisher, the editors and the reviewers. Any product that may be evaluated in this article, or claim that may be made by its manufacturer, is not guaranteed or endorsed by the publisher.
